# *GmNAC06*, a NAC domain transcription factor enhances salt stress tolerance in soybean

**DOI:** 10.1007/s11103-020-01091-y

**Published:** 2020-11-05

**Authors:** Ming Li, Rui Chen, Qiyan Jiang, Xianjun Sun, Hui Zhang, Zheng Hu

**Affiliations:** 1 Zhengzhou Fruit Research Institute, Chinese Academy of Agricultural Sciences, Zhengzhou, 450009 China; 2 National Key Facilities for Crop Genetic Resources and Improvement, Institute of Crop Sciences, Chinese Academy of Agricultural Sciences, Beijing, 100081 China; 3 Biotechnology Research Institute, Tianjin Academy of Agricultural Sciences, Tianjin, 300192 China

**Keywords:** Soybean, NAC, Hairy roots, CRISPR-Cas9, Salt tolerance

## Abstract

**Key message:**

We found *GmNAC06* plays an important role in salt stress responses through the phenotypic, physiological and molecular analyses of OE, VC, and Mutant composite soybean.

**Abstract:**

Salinization affects 20% of all cultivated land worldwide because of the high salinity of irrigation water and the excessive use of water, and this amount is increasing daily. NAC (NAM, ATAF, and CUC) have been found to be involved in salt stress. In this study, a soybean NAC gene, *GmNAC06* (Glyma06g21020.1), was cloned and functionally characterized. The results of expression analysis suggested that salt stress could influence the expression level of *GmNAC06*. The subcellular localization analysis results suggested that *GmNAC06* may function as a transcription factor. Under salt stress, the overexpression technology combined with CRISPR-Cas9 system found that *GmNAC06* could cause the accumulation of proline and glycine betaine to alleviate or avoid the negative effects of ROS; similarly, it could control the Na^+^/K^+^ ratios in hairy roots to maintain ionic homeostasis. The fresh weight of the transgenic hairy roots and the histochemical ROS staining of wild leaves suggested that transgenic hairy roots influence the function of wild leaves under salt stress conditions. Moreover, the expression levels of *GmUBC2* and *GmHKT1* were higher in the *GmNAC06* hairy roots than in the control. Thus, the overexpression of *GmNAC06* in hairy roots notably causes an entire composite plant to exhibit salt tolerance. The phenotype of composite soybean plants and transgenic Arabidopsis plants suggest that GmNAC06 plays a role in response to salt stress and could be useful in generating salt tolerant transgenic crops.

**Electronic supplementary material:**

The online version of this article (10.1007/s11103-020-01091-y) contains supplementary material, which is available to authorized users.

## Introduction

Soybean (*Glycine max*) is the fourth largest crop in the world. It is an important cash crop for food, fuel and feed, and it has been used as a raw material in human health and industrial products. Therefore, the global demand for soybean is increasing gradually. Soybean production is affected by biotic and abiotic stresses, such as inadequate water supplies, high salinity, and low temperatures. Salinity is a major environmental factor limiting the growth and yield of soybean, and it has a negative influence at the cellular, organ and even whole-plant levels. The growth response to salt stress has two phases (Munns and Tester 2008). During the first, rapid phase, plant growth slows because of osmotic responses to the salt outside the roots, so it is more difficult for the roots to obtain water. Due to the decreased water absorption capacity of roots, the water loss from leaves is accelerated; therefore, salinity stress is also considered hyperosmotic stress (Munns 2005). Next, the growth of the shoots is limited. In addition, the stomata close to protect the plant against the ion flow. Finally, the growth of the young leaves slows. During the second, slower phase, a further decrease in plant growth is observed due to the toxic contents of salt inside the plants. Plants, on the basis of adaptive evolution, can be roughly classified into two major types: halophytes (that can withstand high salinity) and glycophytes (that cannot withstand high salinity and eventually die under saline conditions). Soybean belongs to the second category. Therefore, it is of great significance to study the mechanism of salt tolerance in soybean molecular breeding.

The NAC protein family is one of the largest families of TFs in plants; for example, there are 117 *NAC* genes in *Arabidopsis thaliana* (Nuruzzaman et al. 2010), 152 in tobacco (Rushton et al., 2008), 151 in rice (Nuruzzaman et al. 2010), 152 in maize (Shiriga et al. 2014), and 152 in soybean (Le et al. 2011). NAC proteins consist of two parts. The NAC domain at the N-terminus is conserved, contains 150–160 amino acids and is divided into five subdomains (A–E) (Ooka et al. 2003). Every subdomain has its own function; subdomain A may be responsible for the formation of a functional dimer, subdomains B and E may be involved in the functional diversity of NAC genes, and subdomains C and D are used to bind to DNA (Puranik et al. 2012). The second part is a highly divergent C-terminus transcription regulatory (TR) region that acts as a transcriptional activator or repressor and has frequent occurrences of simple amino acid repeats and regions rich in serine and threonine, proline and glutamine, or acidic residues (Olsen et al. 2005; Puranik et al. 2012). NAC proteins are plant-specific TFs that are relevant to the development of plants (Shen et al. 2009). *NTM1* regulates cell division (Kim et al. 2006). *OsNAC05* influences seed development (Sperotto et al. 2009). The *Arabidopsis* NAC TF *ANAC92* has been shown to control senescence (Balazadeh et al. 2010). *GmNAC20* is involved in the formation of the lateral root (Hao et al. 2011). In addition, numerous NAC genes are involved in responses to abiotic stresses, such as salinity, drought and cold. *OsNAC45* may enhance the salt tolerance of transgenic rice (Zheng et al. 2009). The overexpression of *ZmSNAC01* may enhance tolerance to drought in *Arabidopsis* (Lu et al. 2012). *TaNAC02* increases the cold tolerance of *Arabidopsis* (Mao et al. 2012).

Because of the high salinity of irrigation water and the excessive use of water, more than 800 million hectares of land are affected by salinization globally (Krishnamurthy et al. 2019). Sodium chloride is soluble and widespread, so roots may take up many Na^+^ and Cl^+^, ions that negatively affect the metabolic processes and photosynthetic efficiency of plants (Mäser et al. 2002). The root is the first plant organ to experience salt damage in saline soil, so it is important to study the salt tolerance of roots. Although the transgenic soybean has been cultivated at a large scale in the world, its genetic transformation still presents difficulties. Hinchee et al. (1988) obtained transgenic soybean plants using the *Agrobacterium tumefaciens*-mediated DNA transfer. Subsequently, some laboratories have studied *A. tumefaciens*-mediated plant transformation, but few bacterial strains are widely used. Through *A. tumefaciens*-mediated transformation of soybean has been greatly improved over the years (Chen et al. 2018). However, it still presents several unresolved problems such as low transformation efficiency, complex procedure, susceptibility to contamination, and a long time period (Pareddy et al. 2020). However, genetic transformation technology of hairy root can solve these problems. The *A. rhizogenes* cucumopine strain K599 (pRi2659) was isolated and characterized because of an outbreak of hairy root disease in cucumber in the 1970s (Weller et al. 2004; Mankin et al. 2007). After K599 is injected into the cotyledonary node, it may effectively induce hairy roots at the infection site and form composite soybean plants consisting of wild-type shoots and transgenic roots. There are many advantages to the *A. rhizogenes*—mediated transformation system; for example, the experimental procedure is simple, the experiment period is short, and it can be used to study root specific genes. In addition, the transformation efficiency is high. Estrada-Navarrete et al. (2006) tested four strains of *A. rhizogenes* to induce hairy roots on four species of *Phaseolus* and found that K599 was the most effective, with transformation frequencies up to 90%. Kereszt et al. (2007) reported that they had induced hairy roots in soybean with K599, with transformation frequencies of 25–80%. Therefore, *A. rhizogenes* could be used as a tool in functional genomics. Curtin et al. (2011) studied targeted mutations induced through zinc-finger nucleases in soybean hairy roots. Cao et al. (2011) reported that the overexpression of *TaNHX2* in hairy roots enhanced the salt tolerance of the composite soybean. Wang et al. (2015) found that *GmWRKY27* improved salt and drought tolerance in transgenic soybean hairy roots through overexpression and RNAi analysis. Hairy roots were also used to study targeted mutagenesis caused by the CRISPR-Cas9 system (Sun et al. 2015).

To elucidate the role of *GmNAC06* in soybean plants under salt stress, we constructed composite plants though overexpression and CRISPR-Cas9 technology. The physiological parameters, molecular experiment and phenotypic analysis suggested that the overexpression of *GmNAC06* in hairy roots enhances the salt tolerance of the composite plants. The transgenic *Arabidopsis* showed a similar result. However, the salt tolerance of the composite plants edited by the CRISPR-Cas9 system decreased. Based on the results, it is suggested that *GmNAC06* is an ideal candidate gene for enhancing salt tolerance of soybean.

## Materials and methods

### Subcellular localization and transactivation activity analysis

The ORF of *GmNAC06* with a mutational stop codon was cloned upstream of the green fluorescent protein (GFP) in a 16318hGFP vector with the Quick-Fusion Cloning Kit (biotool.cn, Basel, Switzerland) and a pair of primers (Table S1). The recombinant *35S::GmNAC06:GFP* and a control plasmid *35S::GFP* (10 μg) were transformed into *Arabidopsis* leaf protoplasts as described previously (Wang et al. 2017). 158 amino acids in the N-terminal of *AtBZR2* (AT1G19350.3) contained a nuclear localization signal (NLS), which were fused with mCherry as nuclear marker (Zhang et al. 2016). After 18 h, the GFP fluorescence was observed under a laser scanning confocal microscope (Olympus FV1000 viewer, Tokyo, Japan).

To perform the transactivation activity in yeast cells, the ORF of *GmNAC06* was cloned into pGBKT7 with the Quick-Fusion Cloning Kit and a pair of primers (Table S1). pGBKT7-AtDREB2A was the positive control, while the pGBKT7 empty vector was the negative control (Sakuma et al. 2006). The Yeastmaker™ Yeast Transformation System 2 (Clontech, United States) was used for yeast transformation.

### Construction of soybean and *Arabidopsis* transgenic plants

The TRIzol reagent (Invitrogen, Carlsbad, United States) was used to isolate Total RNA from soybean (*Glycine max* L. Merr.) cultivar Williams 82. According to the instructions, genomic DNA was removed by DNase I (Thermo Scientific, Waltham, United States). The first-strand complementary DNA (cDNA) synthesis was performed with the RevertAid First Strand cDNA Synthesis Kit (Thermo Scientific, Waltham, United States). The primers (Table S1) and Pfu DNA polymerase (TransGen Biotech, Beijing, China) were used to get 1074 bp full-length open reading frames (ORF) of *GmNAC06*. The products were purified and integrated into the blunt vector (*pEASY*-Blunt Simple Cloning Kit, Beijing, China) for sequencing. *GmNAC06* was cloned into pCAMBIA3301 with the Quick-Fusion Cloning Kit and a pair of primers (Table S1). pUC57-GmU6-10-sgRNA and pCambia3301-Cas9 were used to construct pCas9-GmU6-sgRNA recombinant vector as previously described (Sun et al. 2015). After sequence verification, the recombinant plasmid and pCAMBIA3301 were transformed into *A. rhizogenes* K599 as overexpression (OE), vector control (VC) and Mutant respectively.

The soybean seeds were placed into identical pots containing mixed soil (humus: vermiculite = 2:1) at a depth of 1–2 cm. The seeds were placed in a greenhouse at 28 °C and watered daily. The 6-day-old seedlings with folded cotyledons (Fig. S3a) were infected by *A. rhizogenes* strain K599 with the OE, VC and Mutant vector around cotyledonary node area using syringe needle (Fig. S3b and c). The soybean was kept in a 12 h light/12 h dark cycle at 28 °C. After infection, seedlings were covered with a transparent lid (Fig. S3d). After the initiation of the hairy root formation from the wounding sites, the infection sites and parts below it were covered by vermiculite to maintain a high humidity (Fig. S3e). Four weeks later, when the hairy roots were approximately 5–10 cm and could support the plant (Fig. S3f and g), the main roots were removed (Fig. S3h). The OE hairy roots were screened by RT-PCR and qRT-PCR (Fig. S4), the VC hairy roots were screened by PCR analysis of the GUS gene (Fig. S5), T7 endonuclease 1 (T7E1) was used to detect the PCR products of the Mutant hairy roots (Fig. S6), and the original roots were removed from the composite plants. The plants with hairy roots were transferred into mixed soil (humus: vermiculite = 2:1) and watered every 3 days (Fig. S3i).

The overexpression vector and pCAMBIA3301 were transformed into *A. tumefaciens* strain GV3101 as the overexpression (OE) and vector control (VC) and then into wild-type (WT) *Arabidopsis* Columbia ecotype by the floral dip method (Clough and Bent 1998). The phosphinothricin resistance and PCR were used to screen seeds (Fig. S7a and b). Homozygous T_3_-generation plants were used for further analyses.

### Stress treatments and quantitative reverse transcription PCR (qRT-PCR) analysis

Williams 82 was used to analyze tissue-specific expression. RNA was extracted from roots, cotyledons, stems and leaves of 20-day-old soybean plants. The RNA of flowers was isolated from mature plants. The seeds were germinated in pots containing vermiculite in a greenhouse at 28 °C with a 12 h light/12 h dark cycle and 50% relative humidity. The roots of the 20-day-old seedlings were immersed in Hoagland solutions embodying 250 mM NaCl (salt treatment), 20% polyethylene glycol (PEG) (dehydration treatment) and 100 μM ABA (ABA treatment). The 20-day-old seedlings were placed at 4 °C for the the cold treatment. RNA was isolated from the roots and leaves at 0.5 h, 2 h, 6 h, 12 h and 24 h after treatment.

cDNA samples, Maxima SYBR Green/ROX qPCR Master Mix (Thermo Scientific, Waltham, United States), the primers (Table S1) and the Eco Real-Time PCR system (Illumina, San Diego, CA, USA) were used to perform qRT-PCR. All experiments were repeated three times. The data were analyzed with the comparative CT method (2^−△△CT^ method) (Livak and Schmittgen 2001). cDNA samples of *GmNAC06* from the OE and VC hairy roots were used to examine the expression level of 14 salt stress-related genes by qRT-PCR (Table S1). The expression level of **2** salt stress-related genes in the OE, VC and WT Arabidopsis were examined by qRT-PCR (Shkolnik et al. 2013; Sun et al. 2020). The soybean *CYP2* gene and Arabidopsis *UBQ3* gene were used as a reference for normalization (Ni et al. 2013).

After 6 weeks, when the hairy roots had recovered enough healthy, the OE, VC and Mutant composite plants were irrigated with 250 mM NaCl solution. Two days later, the leaves and hairy roots were used to analyze the function of *GmNAC06*. After 2 weeks, thirty OE, thirty VC, thirty Mutant composite plants under normal conditions and same number under salt stress were used to analyze root growth phenotype. After the salt treatment, thirty OE, thirty VC and thirty Mutant composite plants were used to determine the salt damage index (SDI%) for 2 consecutive weeks (Cao et al. 2011).

Seeds of the VC (VC3), OE6, OE9 and OE17 plants of the homozygous T_3_ generation were surface-sterilized and planted on MS medium without NaCl or with 100 mM NaCl. After 3 days of vernalization at 4 °C, the percentage germination (%) was measured for 1 consecutive week. Each sample contained 36 seedlings, and the experiments were replicated three times. To evaluate the root growth under normal conditions and the 100 mM NaCl treatment, 5-day-old seedlings with roots of nearly equal length were placed vertically in a growth chamber. After 7 days of incubation, the root elongation lengths of 30 seedlings were determined. For the salt tolerance assay, the homozygous T_3_ generation was germinated soil chambers in a greenhouse at 22 °C with a 16 h light/8 h dark cycle and 70% relative humidity, and the 4-week-old potted *Arabidopsis* plants were subjected to a 250 mM NaCl treatment. After 2 weeks, the survival rates were scored. Each sample contained 12 seedlings, and the experiments were replicated three times.

### Histochemical and physiological analysis

Under salt stress, some plants can generate hydrogen peroxide (H_2_O_2_) and superoxide (${\text{O}}_{2}^{ - }$) to mediate numerous physiological and biochemical processes. H_2_O_2_ and ${\text{O}}_{2}^{ - }$ were visually detected with 3,3′-diaminobenzidine (DAB) (Beijing Biodee Biotechnology, Beijing, China) and nitro blue tetrazolium (NBT) (Beijing Biodee Biotechnology, Beijing, China). The leaves of the OE, VC and Mutant composite plants were infiltrated with 5 mg/ml DAB at pH 3.8 for 20 h and 0.5 mg/ml NBT at pH 7.5 for 20 h in the dark to detect H_2_O_2_ and ${\text{O}}_{2}^{ - }$. Then, the leaves were transferred to ethanol (glycerol: 95% ethanol = 3:7) and boiled for fifteen minutes in a water bath. After cooling, the leaves were extracted at room temperature with ethanol (glycerol: 95% ethanol = 3:7) until the leaves were decolorized completely (Kong et al. 2011). The programmed cell death was analyzed by trypan blue (Huang et al. 2011). Trypan blue can color dead cells into blue; however, living cells are not stained. The leaves of the OE, VC and Mutant composite plants were soaked in 0.4% trypan blue solution (MYM Biological Technology Company Limited, Chicago, IL, USA), boiled for two minutes in a water bath, and then incubated for 8 h. The leaves were transferred to 1.25 g/ml chloral hydrate solution (MYM Biological Technology Company Limited, Chicago, IL, USA) for fading for 3 days, and the chloral hydrate solution was changed one time every day.

For measuring H_2_O_2_ content (Liu et al. 2010), O_2_^−^ production rates (Elstner and Heupel 1976), MDA content (Puckette et al. 2007), electrolyte leakage (Zhao et al. 2004), glycine betaine content (Li et al. 2017) and proline content (Pinedo et al. 2015), leaf and hairy root sample were taken from three different plants and experiments were replicated three times. The Na^+^ and K^+^ contents were measured by using an inductively coupled plasma optical emission spectrometer (ICP-OES, United States).

The chlorophyll content was measured in the leaves of the OE and VC *Arabidopsis* plants before the salt stress treatment and 3 days after the salt stress treatment. Each leaf sample came from three different plants. The chlorophyll were extracted by acetone (80%) and measured absorbances at 663 and 645 nm by Beckman DU800 spectrophotometer (Fullerton, CA, United States) (Aono et al. 1993). All experiments were replicated three times.

### Statistical analysis

All experiments were replicated independently at least three times, and data are shown as the mean ± SD of three independent experiments. Data were subjected to analysis of variance (ANOVA) using the Statistical Analysis System (SPSS version 22.0) software. The differences between the means were compared using the Tukey's test (P < 0.05).

## Results

### Characterization of *GmNAC06*

A comparison of the amino acid sequences of the N-terminal subdomains showed that *GmNAC06* proteins are 32.13–55.47% identical to *Arabidopsis AtNAC* proteins. Subdomains A, C and D are highly conserved, and subdomains B and E are divergent (Fig. S1a). The phylogenetic tree was constructed using MEGA 6. A neighbor-joining evolutionary phylogeny test and 500 bootstrap replicates were selected for the analysis. The result suggested that *GmNAC06* clustered with *AtNAC*2 (Fig. S1b).

### Subcellular localization and transactivation activity analysis of *GmNAC06*

To determine the subcellular localization of *GmNAC06*, the *35S::GmNAC06:GFP* fusion protein (Fig. S2a) and control *35S::GFP* were transferred into the *Arabidopsis* protoplast. *AtBZR2* fused to mCherry as nuclear marker. The control *35S::GFP* distributed throughout the whole cell, whereas the *35S::GmNAC06:GFP* fusion protein was detected only in the nucleus of the *Arabidopsis* protoplast (Fig. S2b). This result suggests that *GmNAC06* may function as a transcription factor.

In order to test for transactivation activity of GmNAC06, the fusion plasmids pGBKT7-GmNAC06, the positive control pGBKT7-AtDREB2A, and the negative control pGBKT7 were separately transformed into yeast strain AH109. The transformed yeast strain grew well in non-selective medium SD/–Trp and, using X-α-Gal, it was observed that both the positive control and the cells harboring pGBKT7-GmNAC06 displayed β-galactosidase activity, whereas the negative control exhibited no β-galactosidase activity (Fig. S2d), suggesting that GmNAC06 possesses transcriptional activation activity in yeast cells.

### Tissue-specific and stress-responsive expression of *GmNAC06*

**Fig. 1:**
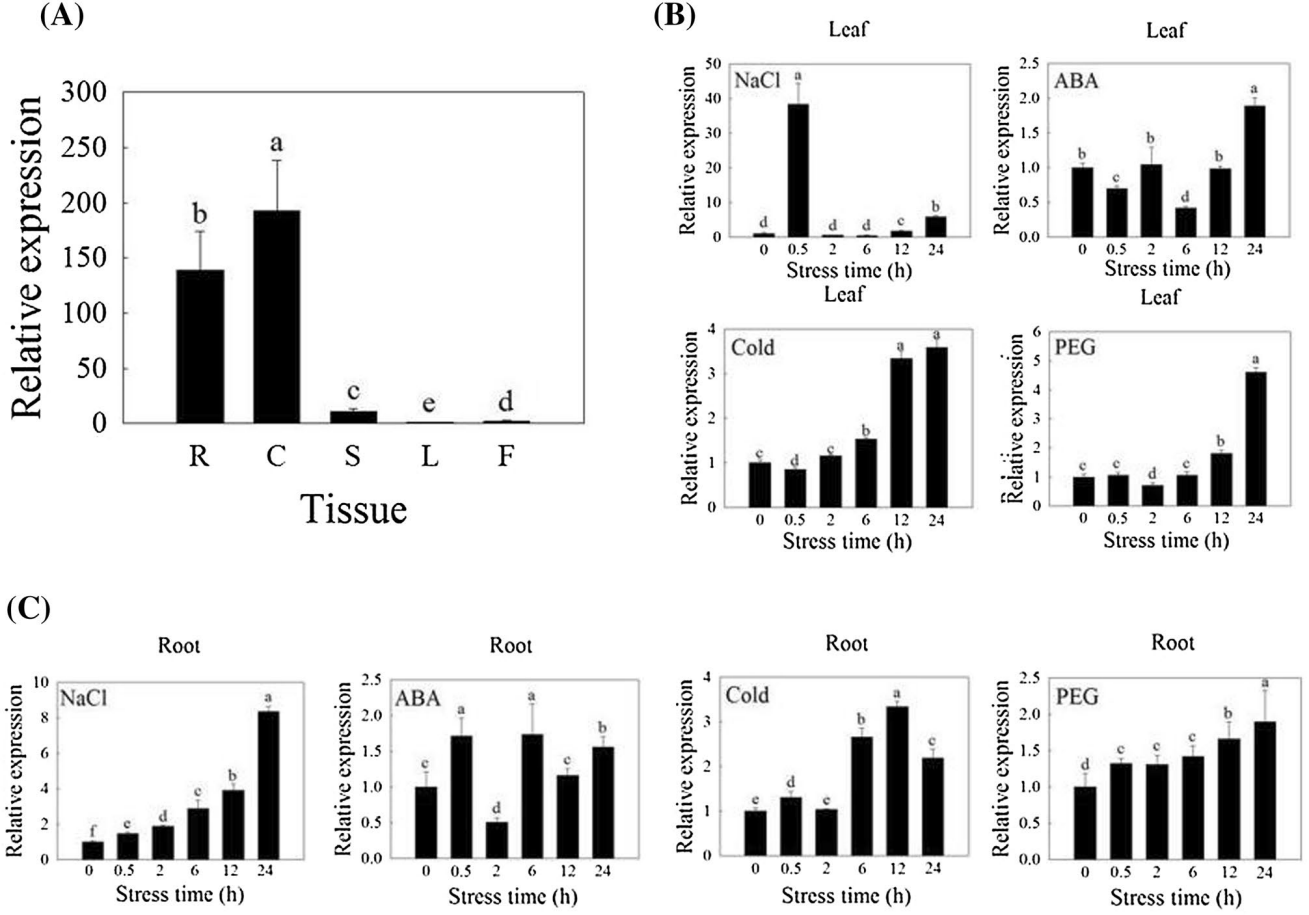
The transcription level of *GmNAC06* in various organs and in response to various stresses. **a** The transcription level of *GmNAC06* in different soybean organs was measured by qRT-PCR. **b** Leaf samples and **c** root samples under various stresses were collected at different time points to analyze the expression patterns of *GmNAC06* by qRT-PCR. The values represent the averages of three independent biological experiments, and the error bars represent standard deviations. Different letters represent significant differences (*P* < 0.05)

The different soybean organs were collected to analyze the tissue-specific expression of *GmNAC06* by qRT-PCR (Fig. 1a). The results showed *GmNAC06* was expressed constitutively in soybean. The transcription levels of *GmNAC06* in roots were 0.72, 12.97, 138.76 and 59.72 times that of cotyledons, stems, leaves and flowers, respectively.

To learn the gene function of *GmNAC0*6, leaf and root samples under various stresses were collected at different time points to analyze the transcription level of *GmNAC06* by qRT-PCR (Fig. 1b, c). In the leaves under NaCl stress, the transcriptional level of *GmNAC06* at 0.5 h after treatment was 38.4 times that of 0 h. Under the ABA treatment, the transcriptional level of *GmNAC06* at 6 h after treatment was 0.4 times that of 0 h. Under the cold and PEG stresses, the transcriptional level of *GmNAC06* at 24 h after treatment was 3.6, 4.6 times that of 0 h, respectively. In the roots under NaCl stress, the transcriptional level of *GmNAC06* at 24 h after treatment was 8.4 times that of 0 h. Under the ABA treatment, the transcriptional level of *GmNAC06* at 2 h after treatment was 0.5 times that of 0 h. Under cold stress, the transcriptional level of *GmNAC06* at 12 h after treatment was 3.3 times that of 0 h. Under PEG stresses, the transcriptional level of *GmNAC06* at 24 h after treatment was 1.9 times that of 0 h. It suggested that *GmNAC06* in the leaf and root responds to salt, exogenous ABA, cold and PEG stresses.

### Effect of GmNAC06 overexpression on salt stress tolerance of composite plants

**Fig. 2:**
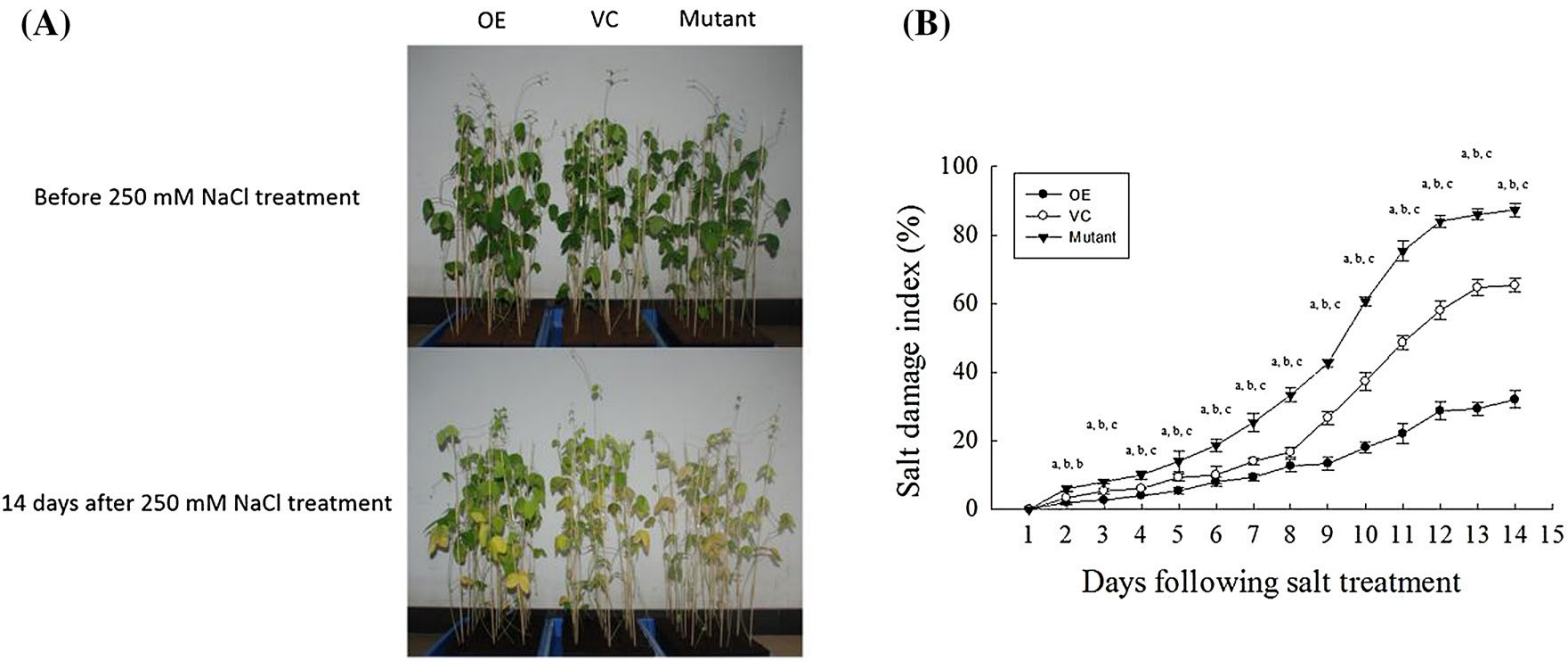
Salt tolerance of composite plants. **a** The 6-week-old composite plants (OE, VC, Mutant) were irrigated with 250 mM NaCl three times a week for 2 consecutive weeks. The photographs were taken 2 weeks after the salt treatment. **b** The difference in the SDI (%) among the composite plants with the OE, VC and Mutant hairy roots were recorded every day for 2 consecutive weeks. Different letters represent significant differences (*P* < 0.05)

Four weeks after the seedling was infected by *A. rhizogenes* K599 with the OE, VC and Mutant vectors, the positive hairy roots of the OE, VC and Mutant composite plants were screened, and then the main roots and original roots were removed. After 6 weeks, the composite plants were irrigated with 250 mM NaCl solution three times a week for 2 consecutive weeks (Fig. 2a). After salt treatment, thirty OE individual plants, thirty VC individual plants and thirty Mutant individual plants were used to record the salt damage index (SDI%) for 2 consecutive weeks (Fig. 2b). Fourteen days after the salt stress treatment, the SDI of the VC and Mutant composite plants were 2 times and 2.7 times compared with the OE composite plants, respectively. The OE composite plants displayed salt tolerance, the VC composite plants wilted and died, and 26 Mutant composite plants dead. These results showed that the overexpression of *GmNAC06* in hairy roots can enhance the salt tolerance of composite plants.

### Effect of GmNAC06 overexpression on salt stress tolerance of hairy roots

**Fig. 3:**
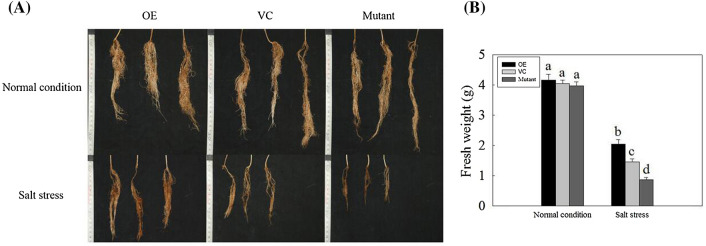
The hairy root growth phenotypes and fresh weights of the composite plants (OE, VC, Mutant) at normal conditions and under salt stress. **a** To determine the phenotypes of hairy roots, thirty samples were analyzed, and the typical samples were selected. **b** The fresh weight of the OE, VC, and Mutant hairy roots under normal conditions and salt stress. Different letters represent significant differences (*P* < 0.05)

Six-week-old composite plants (OE, VC, Mutant) with positive hairy roots were subjected to salt stress. As a control, composite plants were planted in normal conditions. Two weeks later, photographs of the hairy roots were taken (Fig. 3a). Thirty individual hairy roots of OE, VC and Mutant under normal conditions and salt stress were used to calculate the fresh weight (Fig. 3b). The hairy roots of the OE, VC and Mutant composite plants showed no obvious differences when they were cultivated under normal conditions. Under salt stress, the fresh weight of OE, VC and Mutant hairy roots were 49%, 36% and 22% of that under normal conditions, respectively. These results suggest that *GmNAC06* can enhance the salt tolerance of hairy roots.

### Effect of GmNAC06 overexpression on ROS production in composite plants

**Fig. 4:**
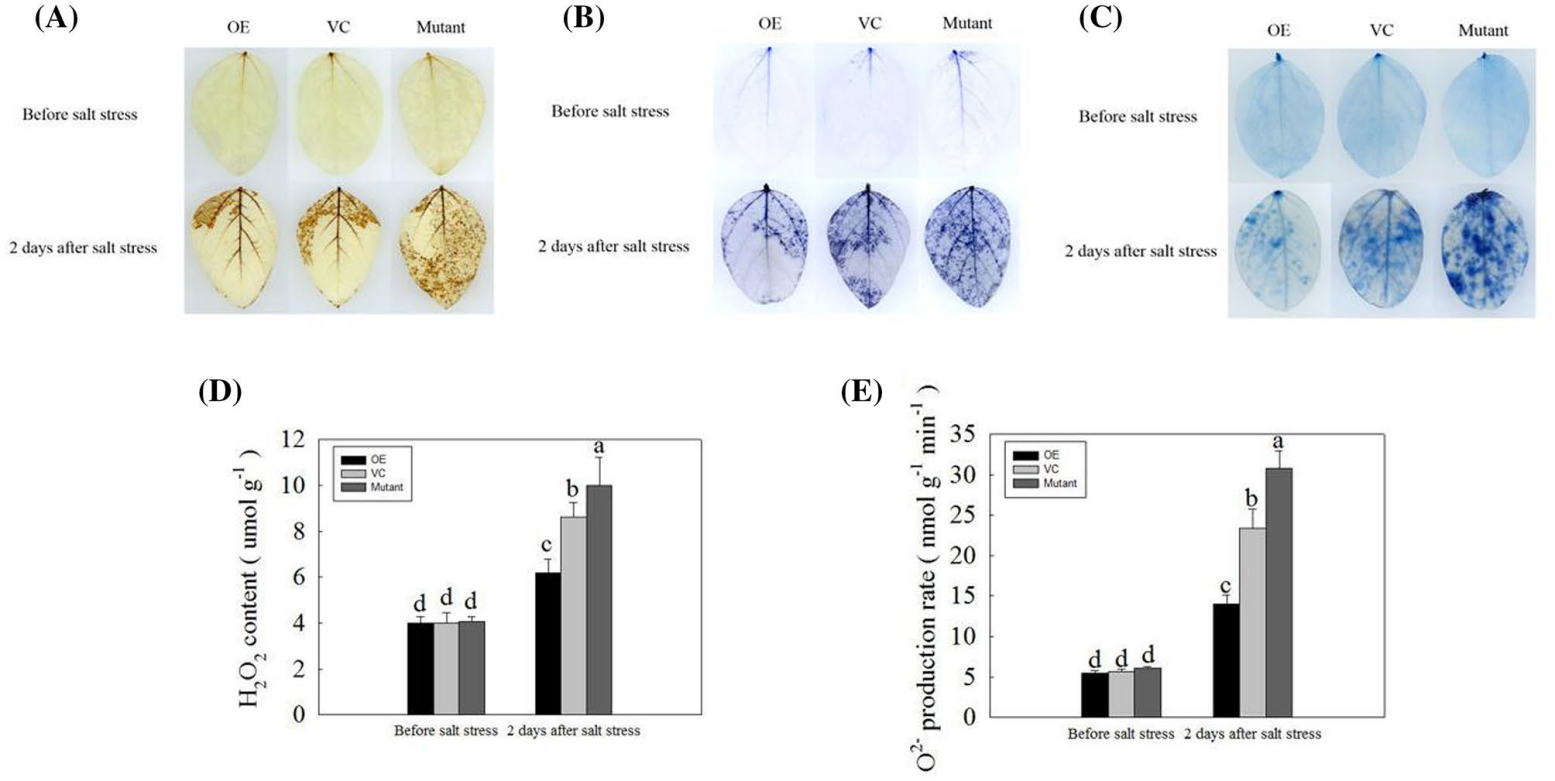
ROS accumulation and cell death in composite plants before salt stress and 2 days after salt stress. The leaves of the composite plants (OE, VC, Mutant) before salt stress and 2 days after salt stress were stained by **a** DAB, **b** NBT, and **c** trypan blue. The **d** H_2_O_2_ content and **e** O_2_^−^ production rate of the composite leaves (OE, VC, Mutant) before salt stress and 2 days after salt stress. Different letters represent significant differences (*P* < 0.05)

To further investigate the function of *GmNAC06* in regulating salt tolerance, the leaves of 6-week-old composite plants (OE, VC, Mutant) before the salt stress treatment and 2 days after the salt stress treatment were stained by DAB and NBT. The leaves of the composite plants had no obvious differences under normal conditions. However, we found lower brown (Fig. 4a) and blue (Fig. 4b) staining intensities in the VC leaves than the Mutant leaves after the salt stress treatment, and the OE leaves had the lowest levels of brown precipitate and blue spots. The H_2_O_2_ content (Fig. 4d) and O_2_^−^ production rate (Fig. 4e) of the Mutant leaves were higher than those of the VC leaves, and both parameters were the lowest in the OE leaves. This result shows that the overexpression of the *GmNAC06* gene in composite plants could reduce ROS production during salt stress. The cell death result is consistent with ROS damage (Fig. 4c). These results suggest the overexpression of the *GmNAC06* gene could enhance the salt tolerance of composite plants.

### Physiological effects of GmNAC06 overexpression in composite plants

**Fig. 5:**
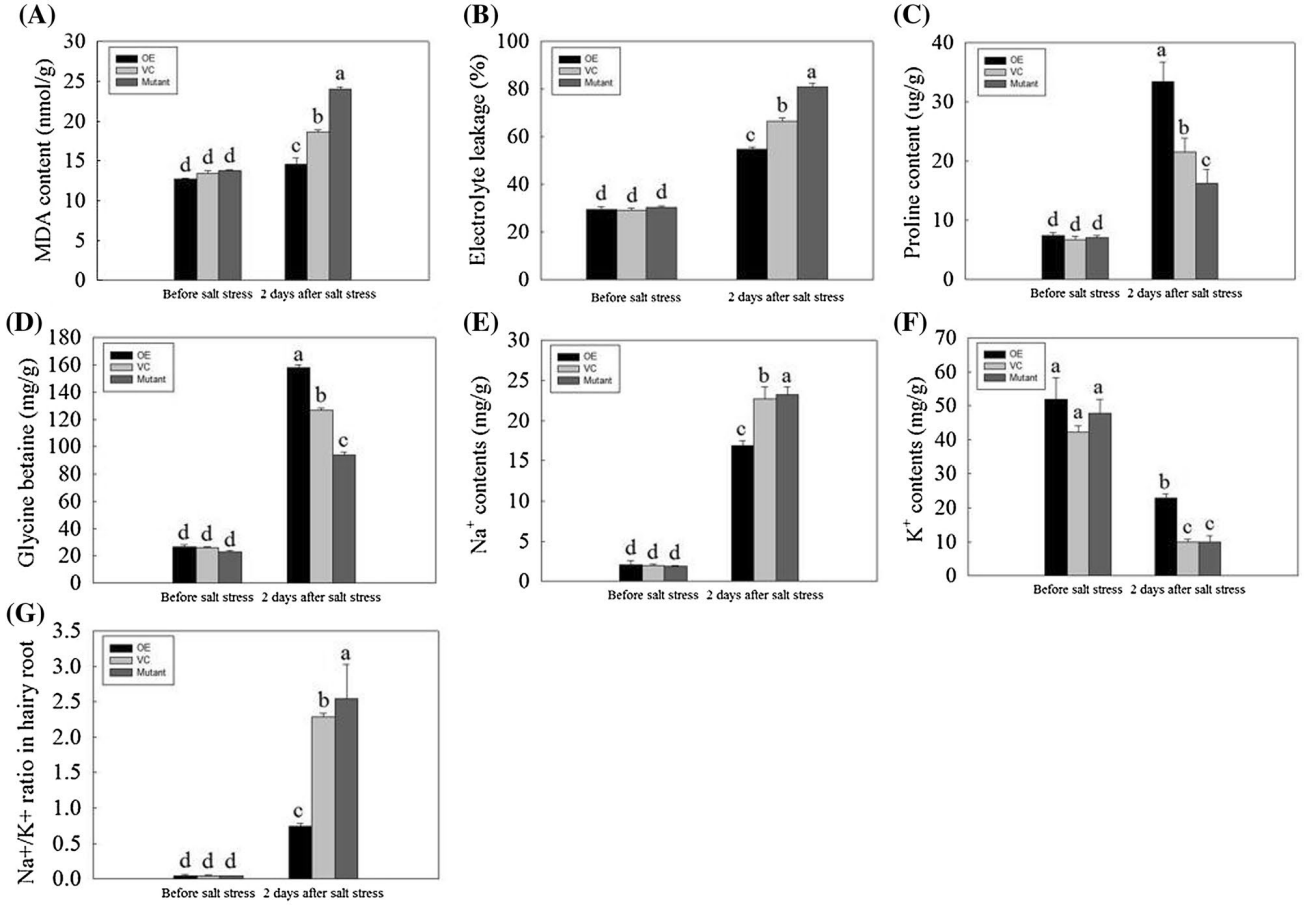
Physiological changes in composite plants before salt stress and 2 days after salt stress. The **a** MDA content, **b** electrolyte leakage, **c** proline content, **d** glycine betaine content, **e** Na^+^ content, **f** K^+^ content and **g** Na^+^/K^+^ ratio of hairy roots in the composite plants before salt stress and 2 days after salt stress. Different letters represent significant differences (*P* < 0.05)

The MDA content is used to suggest lipid peroxidation caused by salt stress (Cao et al. 2007). After salt stress, the MDA content of the hairy roots significantly increased (Fig. 5a). Compared with VC hairy roots, the content of MDA in OE hairy roots decreased by 22%, and that of Mutant hairy roots increased by 29%. Electrolyte leakage is used to suggest the damage caused by salt stress (Zhao et al. 2014). After salt stress, the electrolyte leakage of the hairy roots increased notably. Compared with VC hairy roots, the electrolyte leakage in OE hairy roots decreased by 18%, and that of Mutant hairy roots increased by 22%. (Fig. 5b). Proline and glycine betaine are multifunctional osmolytes, and their accumulation enhances plant stress tolerance, particularly in response to salt stress (Ashraf and Foolad 2007). The proline and glycine betaine accumulation rate among the OE, VC, and Mutant composite plants have no significant difference before salt stress; however, under salt stress, the accumulation of proline and glycine betaine in OE hairy roots increased by 55% and 24% and the accumulation of proline and glycine betaine in Mutant hairy roots decreased by 24% and 26% compared with VC hairy roots (Fig. 5c, d). Maintaining Na^+^ and K^+^ homeostasis is very important for enhancing the salt tolerance of composite plants (Mostofa et al. 2015). Before salt stress, the Na^+^ and K^+^ contents and the Na^+^/K^+^ ratio of the OE, VC, and Mutant hairy roots showed no obvious differences. After salt stress, the Na^+^ content in OE hairy roots decreased by 26%, and that of Mutant hairy roots increased by 3% compared with VC hairy roots (Fig. 5e). However, the K^+^ content in OE hairy roots increased by 129%, and that of Mutant hairy roots decreased by 2% compared with VC hairy roots (Fig. 5f). As a result, the Na^+^/K^+^ ratio of OE hairy roots decreased by 67%, and that of Mutant hairy roots increased by 12% compared with VC hairy roots (Fig. 5g). We detected the expression of *GmNAC06* in the hairy root under normal condition and salt stress (Fig. S6c). Compared with VC hairy roots, the expression level of *GmNAC06* of OE hairy roots increased by 496% and 300%, the expression level of *GmNAC06* of Mutant hairy roots decreased by 63% and 82% under normal condition and salt stress, respectively. Taken together, the results suggest that the overexpression of *GmNAC06* could enhance the salt stress tolerance of composite plants.

### Expression analysis of salt-responsive genes in the OE and VC hairy roots of composite plants

**Fig. 6:**
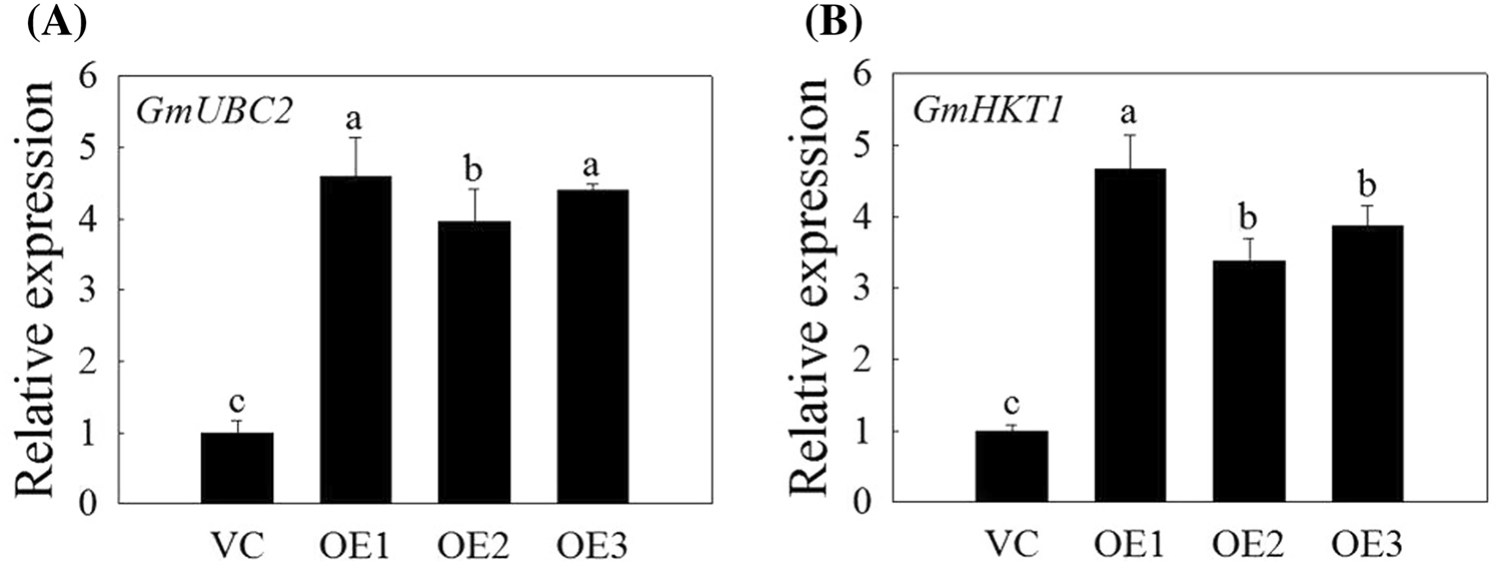
Expression level of salt-related marker genes in the hairy roots of OE and VC composite plants under normal conditions. **a** The transcription level of *GmUBC2*. **b** The transcription level of *GmHKT1*. Each hairy root sample came from three different plants. All experiments were repeated three times. Different letters represent significant differences (*P* < 0.05)

To further understand the function of *GmNAC06*, the expression levels of the 14 salt-related marker genes were analyzed under normal conditions. The expression levels of *GmUBC2* and *GmHKT1* in the OE hairy roots were more than three times higher than those in the control (Fig. 6a, b). This suggests that *GmNAC06* may enhance the salt tolerance of composite plants by regulating salt stress-related genes.

### Effects of overexpression of GmNAC06 in *Arabidopsis*

**Fig. 7:**
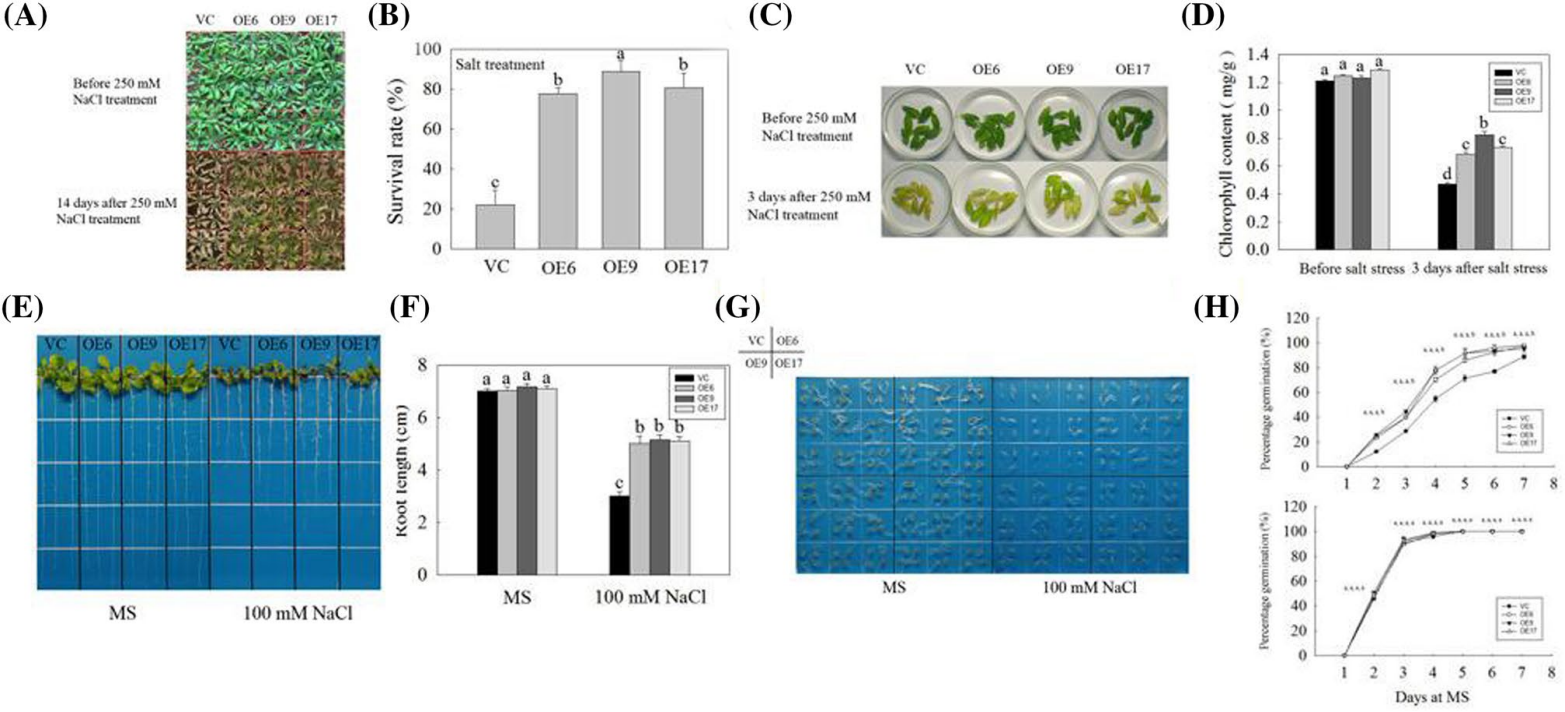
The phenotype and physiological indexes of transgenic *Arabidopsis*. **a** The phenotypes of OE and VC *Arabidopsis* before salt stress and 14 days after salt stress. **b** Survival rate after salt stress. The **c** phenotypes and **d** chlorophyll content of OE and VC *Arabidopsis* before salt stress and 3 days after salt stress. **e** Root growth status, **f** root length, **g** emergence performance and **h** emergence rate of OE and VC *Arabidopsis* grown for 7 days on MS media with 0 or 100 mM NaCl. Different letters represent significant differences (*P* < 0.05)

Homozygous T_3_-generation *Arabidopsis* were used to further analyze the function of *GmNAC06* in salt stress responses. Four-week-old seedlings were treated with 250 mM NaCl solution. Fourteen days after the salt stress treatment, the growth status of the three OE lines was better than that of the VC line (Fig. 7a). The survival rate of the three OE lines was more than three times higher than that of the VC line (Fig. 7b). The leaves of OE and VC *Arabidopsis* were incubated in 250 mM NaCl solution for 3 days (Fig. 7c). After the salt stress, the green of the VC line leaves faded, but leaves of three OE lines were green. The chlorophyll content was measured in leaves of OE and VC *Arabidopsis* before salt stress and 3 days after salt stress (Fig. 7d). Before salt stress, the chlorophyll contents of the OE and VC transgenic lines were not obviously different. However, the chlorophyll content of three OE lines was more than 1.5 times higher than that of the VC line under salt stress. Five-day-old seedlings with roots of nearly equal length were placed vertically on MS solid medium with or without NaCl. After 7 days, the root length of the OE line was similar to that of the VC line under normal conditions. However, the root length of the OE line was more than 1.5 times longer than that of the VC line under salt stress (Fig. 7e, f). To determine whether the three OE lines enhanced the salt tolerance of *Arabidopsis*, seeds from the three OE lines and the VC line were germinated on MS solid medium with or without NaCl. After 3 days of stratifications, the percentage germination was measured for 1 consecutive week. The percentage germination of the OE and VC transgenic lines were not different under normal conditions. Under salt stress conditions, the percentage germination of *Arabidopsis* was inhibited. However, the percentage germination of the three OE lines was higher than that of the VC line under salt stress (Fig. 7g, h). We detected the expression of marker genes *AtUBC2* and *AtHKT1;1* under normal conditions. Their expression levels of in the OE line were more than three times higher than those in the VC and WT Arabidopsis (Fig. S8). Therefore, we concluded that overexpression *GmNAC06* could enhance the salt tolerance of transgenic *Arabidopsis*.

## Discussion

Soybean is considered a salt-sensitive glycophyte, and all of its developmental stages are adversely affected by salinity stress (Phang et al. 2008). Salt stress inflicts heavy losses on soybean yield and has a significant negative impact on the quality. Therefore, it is very important to cultivate salt tolerant soybean varieties. Molecular breeding could improve the efficiency of breeding these varieties. NAC proteins constitute one of the largest families of plant-specific transcription factors, and the NAC protein family is present in a wide range of land plants (Olsen et al. 2005). The bioinformatic data provided by a genome-wide transcriptome analysis predict that 20–25% *NAC* genes function in at least one or more stress responses (Puranik et al. 2012). Increasing evidence has suggested that the NAC family members can enhance salt tolerance in a number of plants, such as *Arabidopsis* (Mao et al. 2014), wheat (Huang et al. 2015), rice (Hong et al. 2016) and pumpkin (Cao et al. 2017). The soybean NAC family contains 152 members. Due to the limitations of soybean transformation technology, the functions of only a few soybean NAC genes have been studied clearly. An *A. rhizogenes*—mediated transformation system could save time and improve the transformation efficiency. A tissue-specific expression assay showed *GmNAC06* was appropriate for an *A. rhizogenes*—mediated transformation system (Fig. 1a, b).

Based on the phylogenetic analysis and sequence alignment, the amino acid sequences of the *GmNAC06* proteins displayed a high similarity to *AtNAC*2 (55.47% identity) (Fig. S1a and b). *AtNAC*2 has been shown to enhance the salt tolerance of groundnut (Patil et al. 2014). The results of qRT-PCR suggested that *GmNAC06* might be involved in stress responses, especially in roots under salt stress, and the expression level increased gradually with time (Fig. 1c). The subcellular localization assay suggested that *GmNAC06* may function as a transcription factor (Fig. S2b). To study the role of *GmNAC06* in salt stress responses, the phenotypes and SDI of OE, VC and Mutant composite plants treated with 250 mM NaCl were observed and recorded (Fig. 2a, b). The results suggested that *GmNAC06* likely functions as a positive stress-responsive transcription factor of salt stress in soybean.

In plants, high concentrations of salt result in physiological damage and water stress, while the water deficit leads to the formation of ROS, and cytotoxic oxygen seriously disrupts vital cellular functions by damaging various cellular components such as proteins, lipids, and DNA (Parida and Das 2005). All these effects can lead to reduced plant growth. Under salt stress, the levels of ROS in plant tissues can be dramatically elevated. Therefore, plants maintaining ROS homeostasis depend on the balance between the generation and scavenging of ROS to be less influenced by the oxidative stress (Jin et al. 2017). The results of the histochemical ROS staining (Fig. 4a, b) and measurement of H_2_O_2_ and ${\text{O}}_{2}^{ - }$ (Fig. 4d, e) showed the OE composite plants could scavenge ROS more effectively than the VC composite plants. The Mutant composite plants accumulated the highest level of ROS because they could not scavenge the ROS in time. The observed occurrence of apoptosis is consistent with the accumulation of ROS (Fig. 4c). Under salt stress, the biomasses of the OE, VC and Mutant hairy roots decreased significantly compared to that of the control (Fig. 3a). However, the OE composite plants showed more developed and faster growing hairy roots compared to the VC composite plants. In contrast, the growth of the Mutant hairy roots suffered serious inhibition (Fig. 3b). This finding suggested *GmNAC06* could enhance the salt tolerance of composite plants by maintaining the normal growth of hairy roots under salt stress. The phenotype of the above ground part of the plant was consistent with the underground part. These results suggested that the function of the wild leaves was influenced by the transgenic hairy roots. MDA content is a typical physiological parameter for evaluating stress tolerance in plants (Yu et al. 2016). It can exhibit the damage degree of membranes. The MDA content of the OE, VC and Mutant hairy roots (Fig. 5a) suggested that overexpression of *GmNAC06* in hairy roots could alleviate cell membrane injury under salt stress. Electrolyte leakage results (Fig. 5b) were consistent with the MDA content. This suggested that increased membrane damage led to increased solute leakage. Proline in plants plays roles in osmotic adjustment, the protection of cellular macromolecules and the scavenging of hydroxyl radicals (Jin et al. 2010). Glycine betaine has an osmoprotective function and is known to improve salt stress tolerance in most crop plants. Glycine betaine enhances the salt tolerance of plants by accelerating of ROS scavenging systems, protecting membrane integrity and reducing the oxidation of membrane lipids under salt stress (Demiral and Türkan 2006). After salt stress, the proline and glycine betaine content of the OE, VC and Mutant hairy roots suggested that *GmNAC06* scavenged ROS by accumulating proline and glycine betaine rapidly to improve the salt tolerance of the hairy roots (Fig. 5c, d). K^+^ is an important macronutrient for plants. Under salt stress, the capacity for K^+^ uptake and transport is usually correlated with the salt tolerance of the plant (Ding et al. 2010). However, Na^+^ could attenuate the intracellular K^+^ influx by disturbing the ion selectivity of cell membranes (Chen et al. 2017). Two methods of salt detoxification include the transport of redundant Na^+^ out the cell through the plasma membrane and the storage of Na^+^ ions in the vacuole (Hasegawa et al. 2000). Plants maintain ionic homeostasis and osmotic equilibrium by preventing Na^+^ influx and promoting K^+^ uptake and Na^+^ extrusion (Zhu 2003). After salt stress, the OE hairy roots maintained a higher K^+^ content and lower Na^+^ content and Na^+^/K^+^ ratio than the VC hairy roots (Fig. 5e–g). Ensuring a low Na^+^/K^+^ ratio in the cytoplasm is very important to improving the salt resistance of plants. All these results suggested the overexpression of *GmNAC06* in hairy roots could help maintain Na^+^ and K^+^ homeostasis. *GmUBC2* had a higher expression level in roots than in stems and leaves. It improved salt tolerance by regulating ion homeostasis, osmolyte synthesis and oxidative stress responses (Zhou et al. 2010). *GmHKT1* enhanced salt tolerance by affecting the Na^+^ and K^+^ transport and regulating the Na^+^/K^+^ homeostasis (Chen et al. 2011). The expression levels of *GmUBC2* and *GmHKT1* in OE hairy roots were significantly higher than control under normal conditions. This suggests that overexpression of *GmNAC06* can improve salt tolerance partly due to the enhanced expression of these genes. Since hairy roots are not heritable, we generated homozygous T_3_-generation *Arabidopsis* to investigate the function of *GmNAC06*. We found that the *GmNAC06*-overexpression transgenic *Arabidopsis* increased salt tolerance compared to the control at both the germination and seedling stages. We further characterized the roles of *GmNAC06* in salt tolerance.

In summary, we found *GmNAC06* plays role in salt stress responses through the phenotypic, physiological and molecular analyses of OE, VC, and Mutant composite soybean. When roots experienced salt stress, overexpression of *GmNAC06* induced the accumulation of proline and glycine betaine to scavenge redundant ROS to protect the integrity of cell membranes. At the same time, it maintained ionic homeostasis and osmotic equilibrium by regulating Na^+^ and K^+^ transport. These measures ensured that the roots could grow normally under salt stress, which improved the salt tolerance of the leaves. Ultimately, the whole composite plants exhibited strong abilities for salt tolerance. These results suggest *GmNAC06* likely functions as a positive regulator of salt tolerance.

## Electronic supplementary material

Electronic supplementary material 1 (JPG 96 kb). Figure S1. Sequence comparison and phylogenetic relationship of *GmNAC06* and NAC family members from *Arabidopsis*. (A) Sequence alignment of the N-terminal subdomains in* GmNAC06* and* Arabidopsis* NAC family members. The* Arabidopsis* Genome Initiative identification numbers of the* Arabidopsis* members are as follows: AtNAC1(AT3G18400.1), AtNAC2(AT5G39610.1), AtNAC3(AT1G76420.1), AtNAC4(AT3G29035.1), AtNAC5(AT1G32770.1), AtNAC6(AT5G62380.1), AtNAC7(AT3G44350.2) AtNAC8(AT5G18270.1), AtNAC9(AT4G10350.1). (B) The phylogenetic tree was constructed using MEGA 6. A neighbor-joining evolutionary phylogeny test and 500 bootstrap replicates were selected for the analysis.

Electronic supplementary material 2 (JPG 100 kb). Figure S2. Subcellular localization and transactivation activity analysis of* GmNAC06*. (A) PCR detection of* 35S::GmNAC06:GFP*. M: DL 2000 maker, 1-8: The amplification of the target fragment by PCR using different liquid bacteria as the template. (B) The vector control (*35S::GFP*) and fusion protein construct 35S::GmNAC06:GFP were introduced into the* Arabidopsis* protoplast. Fluorescent materials were observed under Olympus FV1000 viewer confocal laser scanning microscope, 488 nm, argon-ion laser excitation, 507 nm detection GFP; mcherry was 555 nm, argon-ion laser excitation, LP 640 nm IR detection; chloroplast autofluorescence was 488 nm argon-ion laser excitation, SP 630 nm IR detection, pinhole is about 1.0 unit, and the optical section thickness is about 0.5 µm.* AtBZR2* fused with mCherry was used as a nuclear marker. Scale bars = 10 μm. (C) CDS and peptide sequence of* GmNAC06*. (D) Transactivation activity analysis of* GmNAC06* in yeast cells. pGBKT7-AtDREB2A and pGBKT7 were used as positive and negative controls, respectively.

Electronic supplementary material 3 (JPG 137 kb). Figure S3. Different stages of the soybean hairy root transformation. The ideal stage for transformation: six-day-old seedlings with unfolded cotyledons. (B) Taking the bacterial mass in the petri dish with the tip of a syringe needle. (C) Stabbing the hypocotyl near the cotyledonary node; the red square suggests which part of the seedling had to be infected for a successful transformation. (D) After infection, the seedlings were kept in a plastic tray covered with a transparent lid. (E) After the initiation of hairy root formation from the infection site, the wounding sites and the following were covered with vermiculite to maintain high humidity. (F) Two weeks after inoculation. (G) Four weeks after inoculation. (H) Four weeks after inoculation, during the removal of the main roots (the part below the red line was removed ). (I) The plants with hairy roots were transferred into mixed soil (humus: vermiculite = 2:1) and watered every three days.

Electronic supplementary material 4 (JPG 38 kb). Figure S4.* GmNAC06* overexpression in hairy roots screened by RT-PCR and qRT-PCR. (A) GmNAC06 overexpression in hairy roots screened by RT-PCR. Lanes 1-16: The transcription level of* GmNAC06* in the independent hairy root samples screened by RT-PCR; red represents positive hairy roots, and blue represents negative hairy roots. WT: hairy roots induced by K599 (containing no vector) used as wild-type controls.* CYP2*: internal control. (B) GmNAC06 overexpression in hairy roots screened by qRT-PCR.

Electronic supplementary material 5 (JPG 24 kb). Figure S5. Screened vector control hairy roots. (A) Lanes 1-10: positive hairy roots. WT: PCR products of hairy roots induced by K599 (containing no vector). WT*: PCR products of pCAMBIA3301. M: DL 2000 maker. (B) The transcription level of* GmNAC06* in VC hairy roots by RT-PCR. WT: hairy roots induced by K599 (containing no vector) used as wild-type controls.* CYP2*: internal control.

Electronic supplementary material 6 (JPG 63 kb). Figure S6. CRISPR-Cas9 induced mutations in soybean hairy roots were screened. (A) Detection of mutations using a T7 endonuclease 1 (T7E1) cleavage assay. Lanes 1-15: PCR products amplified from the independent hairy root samples cleaved by T7E1; red represents a mutation, and blue represents no mutation; WT: PCR products of hairy roots induced by K599 (containing no vector) cleaved by T7E1. WT*: PCR products of hairy roots induced by K599 (containing no vector) not cleaved by T7E1. M: DL 2000 maker. (B) Sequence-based detection of mutations by pCas9-GmU6-sgRNA vectors; red represents the protospacer-adjacent motif sequence, and nucleotide substitutions are highlighted in green. +: insertion; -: deletion; S: substitution, X: number of mutations. (C) The expression level of* GmNAC06* under normal condition and salt stress was quantified by qRT-PCR.

Electronic supplementary material 7 (JPG 32 kb). Figure S7. PCR, RT-PCR and qRT-PCR analysis of OE and VC transgenic* Arabidopsis*. (A) Lanes 1-11: The positive lines of OE transgenic* Arabidopsis*. WT: PCR products of OE vector. M: DL 2000 maker. (B) Lanes 1-10: The positive lines of VC transgenic* Arabidopsis*. WT: PCR products of wild* Arabidopsis*. WT*: PCR products of pCAMBIA3301. M: DL 2000 maker. (C) The transcription level of* GmNAC06* in OE and VC transgenic* Arabidopsis* by RT-PCR. UBQ3: internal control. (D) The transcription level of* GmNAC06* in OE and VC transgenic* Arabidopsis* by qRT-PCR.

Electronic supplementary material 8 (JPG 16 kb). Figure S8. Expression level of salt-related marker genes in the OE, VC and WT Arabidopsis under normal conditions. (A) The transcription level of* AtUBC2*. (B) The transcription level of* AtHKT1;1*. Different letters represent significant differences (P < 0.05).

Electronic supplementary material 9 (DOCX 34 kb)
