# Supplementary figures and images for: GmNAC06, a NAC domain transcription factor enhances salt stress tolerance in soybean
Source: Plant Mol Biol. 2020 Nov 5;105(3):333–45. doi: 10.1007/s11103-020-01091-y (PMC7858558; doi:10.1007/s11103-020-01091-y)

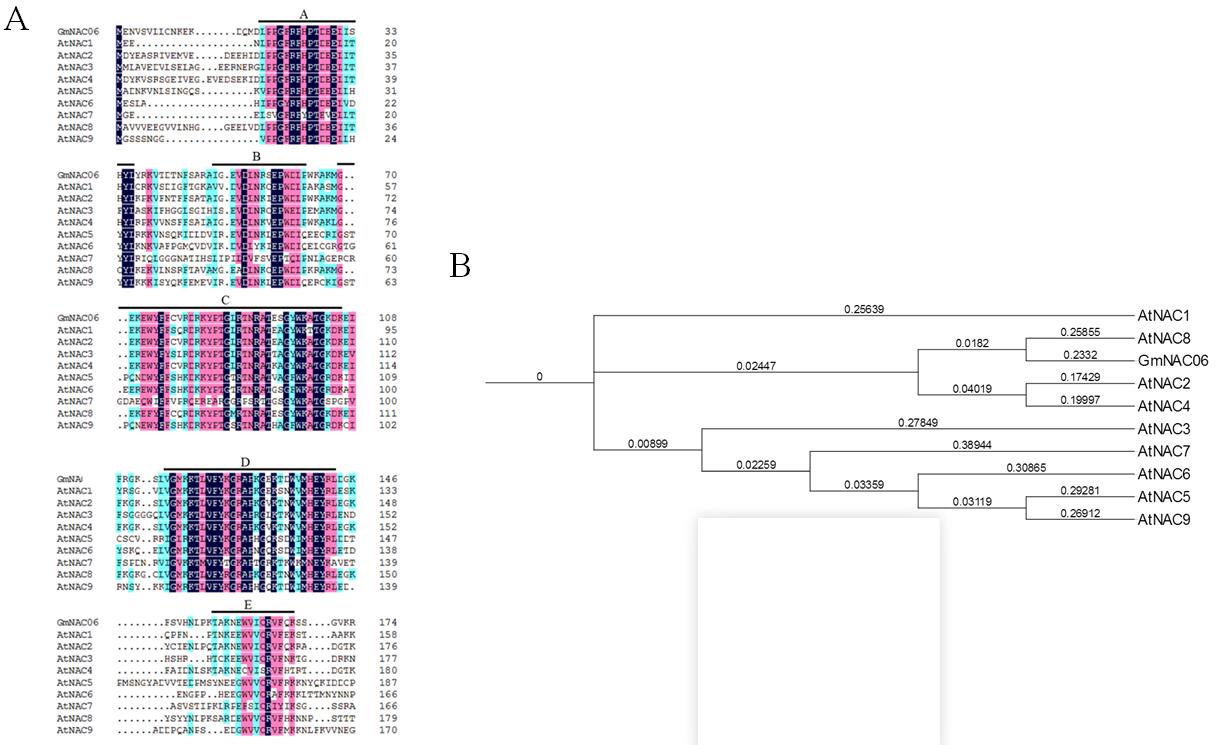

Supplement: Supplementary file 1 — Electronic supplementary material 1 (JPG 96 kb). Figure S1. Sequence comparison and phylogenetic relationship of GmNAC06 and NAC family members from Arabidopsis. (A) Sequence alignment of the N-terminal subdomains in GmNAC06 and Arabidopsis NAC family members. The Arabidopsis Genome Initiative identification numbers of the Arabidopsis members are as follows: AtNAC1(AT3G18400.1), AtNAC2(AT5G39610.1), AtNAC3(AT1G76420.1), AtNAC4(AT3G29035.1), AtNAC5(AT1G32770.1), AtNAC6(AT5G62380.1), AtNAC7(AT3G44350.2) AtNAC8(AT5G18270.1), AtNAC9(AT4G10350.1). (B) The phylogenetic tree was constructed using MEGA 6. A neighbor-joining evolutionary phylogeny test and 500 bootstrap replicates were selected for the analysis. [file 11103_2020_1091_MOESM1_ESM.jpg]

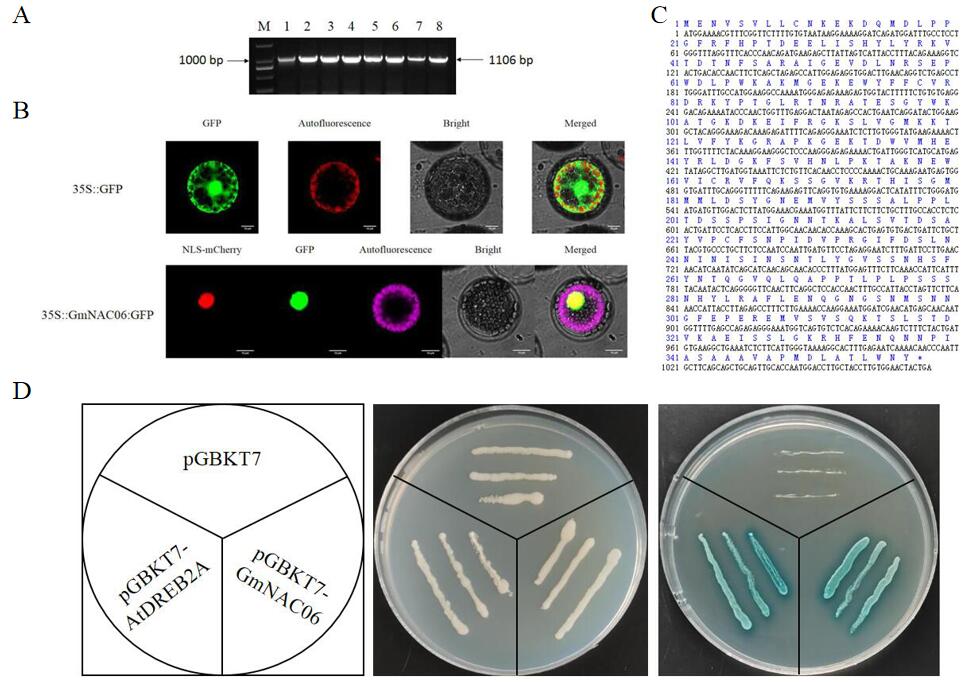

Supplement: Supplementary file 2 — Electronic supplementary material 2 (JPG 100 kb). Figure S2. Subcellular localization and transactivation activity analysis of GmNAC06. (A) PCR detection of 35S::GmNAC06:GFP. M: DL 2000 maker, 1-8: The amplification of the target fragment by PCR using different liquid bacteria as the template. (B) The vector control (35S::GFP) and fusion protein construct 35S::GmNAC06:GFP were introduced into the Arabidopsis protoplast. Fluorescent materials were observed under Olympus FV1000 viewer confocal laser scanning microscope, 488 nm, argon-ion laser excitation, 507 nm detection GFP; mcherry was 555 nm, argon-ion laser excitation, LP 640 nm IR detection; chloroplast autofluorescence was 488 nm argon-ion laser excitation, SP 630 nm IR detection, pinhole is about 1.0 unit, and the optical section thickness is about 0.5 µm. AtBZR2 fused with mCherry was used as a nuclear marker. Scale bars = 10 μm. (C) CDS and peptide sequence of GmNAC06. (D) Transactivation activity analysis of GmNAC06 in yeast cells. pGBKT7-AtDREB2A and pGBKT7 were used as positive and negative controls, respectively. [file 11103_2020_1091_MOESM2_ESM.jpg]

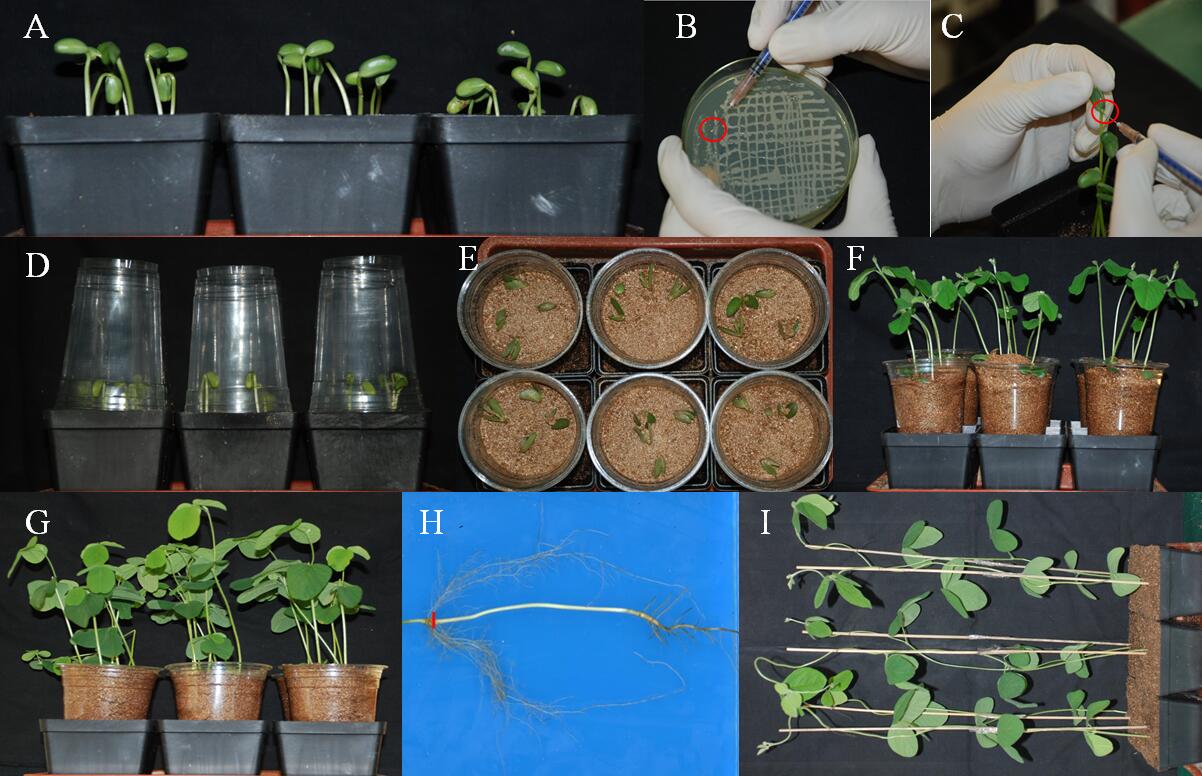

Supplement: Supplementary file 3 — Electronic supplementary material 3 (JPG 137 kb). Figure S3. Different stages of the soybean hairy root transformation. The ideal stage for transformation: six-day-old seedlings with unfolded cotyledons. (B) Taking the bacterial mass in the petri dish with the tip of a syringe needle. (C) Stabbing the hypocotyl near the cotyledonary node; the red square suggests which part of the seedling had to be infected for a successful transformation. (D) After infection, the seedlings were kept in a plastic tray covered with a transparent lid. (E) After the initiation of hairy root formation from the infection site, the wounding sites and the following were covered with vermiculite to maintain high humidity. (F) Two weeks after inoculation. (G) Four weeks after inoculation. (H) Four weeks after inoculation, during the removal of the main roots (the part below the red line was removed ). (I) The plants with hairy roots were transferred into mixed soil (humus: vermiculite = 2:1) and watered every three days. [file 11103_2020_1091_MOESM3_ESM.jpg]

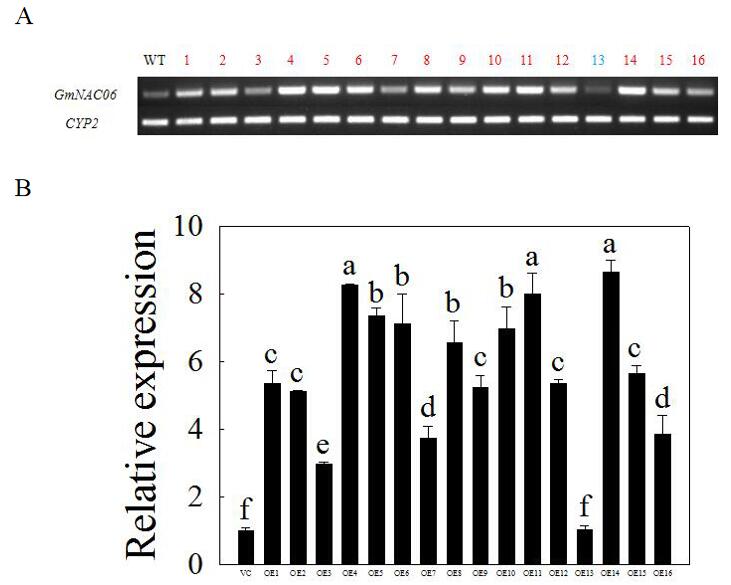

Supplement: Supplementary file 4 — Electronic supplementary material 4 (JPG 38 kb). Figure S4. GmNAC06 overexpression in hairy roots screened by RT-PCR and qRT-PCR. (A) GmNAC06 overexpression in hairy roots screened by RT-PCR. Lanes 1-16: The transcription level of GmNAC06 in the independent hairy root samples screened by RT-PCR; red represents positive hairy roots, and blue represents negative hairy roots. WT: hairy roots induced by K599 (containing no vector) used as wild-type controls. CYP2: internal control. (B) GmNAC06 overexpression in hairy roots screened by qRT-PCR. [file 11103_2020_1091_MOESM4_ESM.jpg]

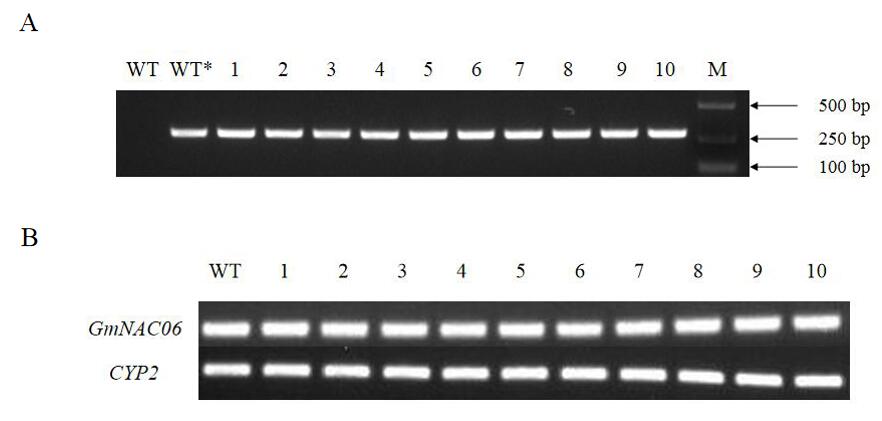

Supplement: Supplementary file 5 — Electronic supplementary material 5 (JPG 24 kb). Figure S5. Screened vector control hairy roots. (A) Lanes 1-10: positive hairy roots. WT: PCR products of hairy roots induced by K599 (containing no vector). WT*: PCR products of pCAMBIA3301. M: DL 2000 maker. (B) The transcription level of GmNAC06 in VC hairy roots by RT-PCR. WT: hairy roots induced by K599 (containing no vector) used as wild-type controls. CYP2: internal control. [file 11103_2020_1091_MOESM5_ESM.jpg]

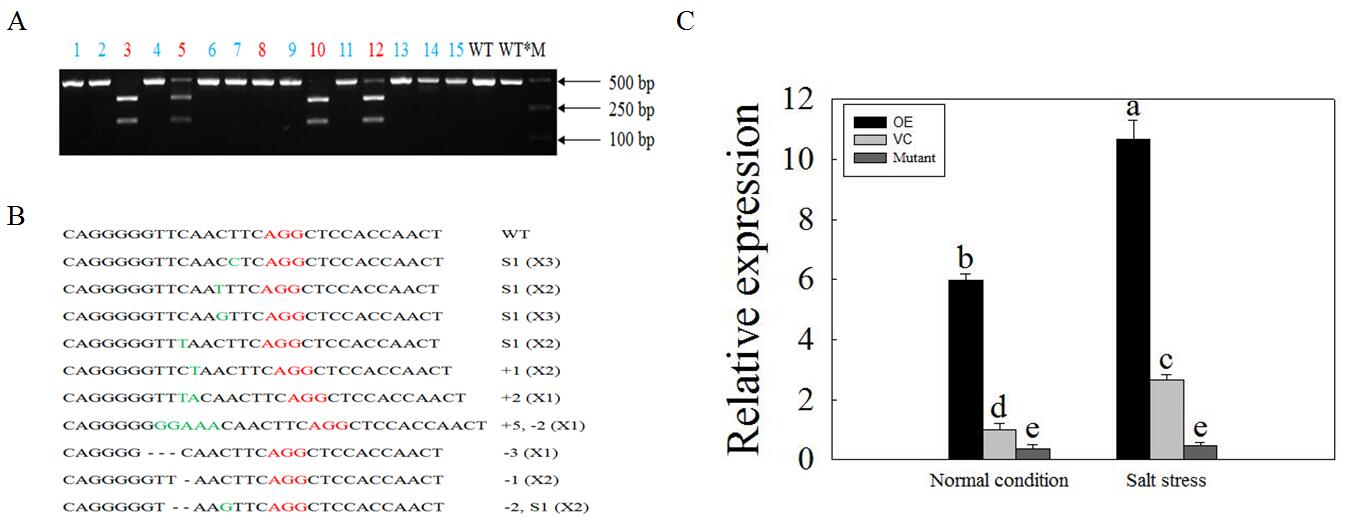

Supplement: Supplementary file 6 — Electronic supplementary material 6 (JPG 63 kb). Figure S6. CRISPR-Cas9 induced mutations in soybean hairy roots were screened. (A) Detection of mutations using a T7 endonuclease 1 (T7E1) cleavage assay. Lanes 1-15: PCR products amplified from the independent hairy root samples cleaved by T7E1; red represents a mutation, and blue represents no mutation; WT: PCR products of hairy roots induced by K599 (containing no vector) cleaved by T7E1. WT*: PCR products of hairy roots induced by K599 (containing no vector) not cleaved by T7E1. M: DL 2000 maker. (B) Sequence-based detection of mutations by pCas9-GmU6-sgRNA vectors; red represents the protospacer-adjacent motif sequence, and nucleotide substitutions are highlighted in green. +: insertion; -: deletion; S: substitution, X: number of mutations. (C) The expression level of GmNAC06 under normal condition and salt stress was quantified by qRT-PCR. [file 11103_2020_1091_MOESM6_ESM.jpg]

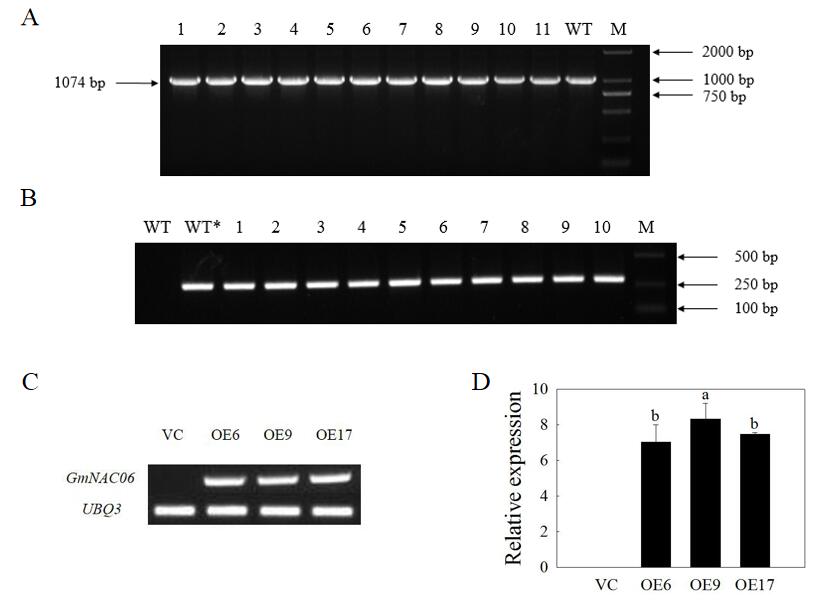

Supplement: Supplementary file 7 — Electronic supplementary material 7 (JPG 32 kb). Figure S7. PCR, RT-PCR and qRT-PCR analysis of OE and VC transgenic Arabidopsis. (A) Lanes 1-11: The positive lines of OE transgenic Arabidopsis. WT: PCR products of OE vector. M: DL 2000 maker. (B) Lanes 1-10: The positive lines of VC transgenic Arabidopsis. WT: PCR products of wild Arabidopsis. WT*: PCR products of pCAMBIA3301. M: DL 2000 maker. (C) The transcription level of GmNAC06 in OE and VC transgenic Arabidopsis by RT-PCR. UBQ3: internal control. (D) The transcription level of GmNAC06 in OE and VC transgenic Arabidopsis by qRT-PCR. [file 11103_2020_1091_MOESM7_ESM.jpg]

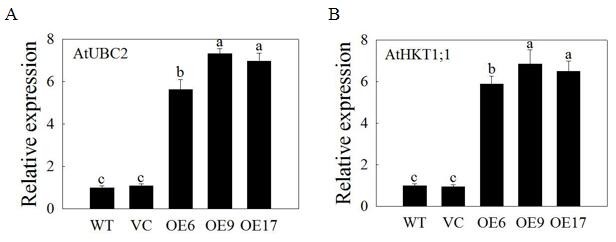

Supplement: Supplementary file 8 — Electronic supplementary material 8 (JPG 16 kb). Figure S8. Expression level of salt-related marker genes in the OE, VC and WT Arabidopsis under normal conditions. (A) The transcription level of AtUBC2. (B) The transcription level of AtHKT1;1. Different letters represent significant differences (P < 0.05). [file 11103_2020_1091_MOESM8_ESM.jpg]
