# Supplementary material for: GmNAC06, a NAC domain transcription factor enhances salt stress tolerance in soybean
Source: Plant Mol Biol. 2020 Nov 5;105(3):333–45. doi: 10.1007/s11103-020-01091-y (PMC7858558; doi:10.1007/s11103-020-01091-y)
Supplement: Supplementary file 9 — Electronic supplementary material 9 (DOCX 34 kb) [file 11103_2020_1091_MOESM9_ESM.docx]

Table S1. List of Primer sequences

| 基因 | 引物 | 引物序列 |
| --- | --- | --- |
| Genes | Primer | Sequence(5'-3') |
| *GmNAC06* | Forward primer | ATGGAAAACGTTTCGGTTCTT |
|  | Reverse primer | TCAGTAGTTCCACAAGGTAGC |
| *GmNAC06-3301* | Forward primer | GAACACGGGGGACTCTTGACATGGAAAACGTTTCGGTTCTT |
|  | Reverse primer | TAGAAATTTACCCTCAGATCTCAGTAGTTCCACAAGGTAGC |
| *GmNAC06-*Crispr | Forward primer | ATTGTACTCAGGGGGTTCAACTTC |
|  | Reverse primer | AAACGAAGTTGAACCCCCTGAGTA |
| *GmNAC06-*GFP | Forward primer | TATCTCTAGAGGATCCATGGAAAACGTTTCGGTTCTT |
|  | Reverse primer | TGCTCACCATGGATCCTCTGTAGTTCCACAAGGTAGC |
| *GmNAC06-*BD | Forward primer | AGGAGGACCTGCATATGATGGAAAACGTTTCGGTTCTT |
|  | Reverse primer | GCCTCCATGGCCATATGTCAGTAGTTCCACAAGGTAGC |
| *GUS* | Forward primer | CGTGTGTCTATGATGATGATG |
|  | Reverse primer | TACGCTGGCCTGCCCAACCTT |
| T7E1 | Forward primer | TTTGCAGGGTTTTTCAGAAGA |
|  | Reverse primer | ATTTCAGCCTTCACATCAGTA |
| *GmNAC06-*qRT-PCR | Forward primer | TGGGATGATGATGTTGGACTCTTAT |
|  | Reverse primer | GGTGTTGCTGTTGATGCTGATAT |
| *GmASN1* | Forward primer | CCCTTTGGTGTTTTGCTC |
|  | Reverse primer | GCCCTTCTTCCTTTTTTATC |
| *GmUBC2* | Forward primer | CTCACATCTATCCAGTCATTGCTTT |
|  | Reverse primer | ACTAAACATTCGAGCTGCTTCA |
| *GmWRKY54* | Forward primer | CCAAGCAGAAGAAGATGATG |
|  | Reverse primer | ACCAGTACTAGAGTTCTCAC |
| *GmWRKY13* | Forward primer | CAGCCGACGGAAAGATCGGC |
|  | Reverse primer | CTTCTTTTTCTTTTCTTTCC |
| *GmWRKY21* | Forward primer | CTTTGCAAGTGACACATATC |
|  | Reverse primer | CGAAACATATAATAACGGTT |
| *GmERF3* | Forward primer | CTTGGACGTTGACTTCGAGGCTGAT |
|  | Reverse primer | AGAGTTAGGCTGCTGCTGGTTGGC |
| *GmMYB76* | Forward primer | GAACAACTGCCAGGGAGAAC |
|  | Reverse primer | TTCATCGGTCCACAAGTCTG |
| *GmMYB92* | Forward primer | GTTGTTCCAAAGTGGGGTTG |
|  | Reverse primer | ACCATCACTTGCAGCCTCTT |
| *GmMYB177* | Forward primer | AGGGGAAAGTGTGGAGCTTT |
|  | Reverse primer | CACTAACCACTGGCGGAACT |
| *GmbZIP1* | Forward primer | GGGGCCCGGGGGTCAAAAGATGAACTTCAG |
|  | Reverse primer | CCCCGGGTCTAGAGGCTTCCTTCGTATAGATGTCA |
| *GmCHI* | Forward primer | GGTCTAGAATGAGAGGTGTTATTGTCTTATT |
|  | Reverse primer | GATACCAAAAGGACAACACTGCCATGGGG |
| *GmST1* | Forward primer | TCTAGAATGGCGTTTGTTGCAGCCATG |
|  | Reverse primer | GAGCTCTCATAAGGTTCGGGGATCCTTTC |
| *GmERF7* | Forward primer | GCGATTATCTCCGACTTCATTC |
|  | Reverse primer | GATTTCACAGTTGTTGCTCCAC |
| *GmHKT1* | Forward primer | TGGTACCGAGTGCAAGACAA |
|  | Reverse primer | ATGGCTGAAGAGATGGAGGA |
| CYP2 | Forward primer | CGGGACCAGTGTGCTTCTTCA |
|  | Reverse primer | CCCCTCCACTACAAAGGCTCG |
| *AtUBC2* | Forward primer | CCAGCGAGGAAGAGATTGAT |
|  | Reverse primer | TGAAAGTACCTCCATCCCAAG |
| *AtHKT1;1* | Forward primer | TCAGTGCATATGGAAACGTTGG |
|  | Reverse primer | CAGCCACCATCGCTGATG |
| *UBQ3* | Forward primer | TGAAAGTACCTCCATCCCAAG |
|  | Reverse primer | TGAAAGTACCTCCATCCCAAG |
